# Preparation of Monolithic Silica Nanocomposite Plates Embedding Fluorescent Graphene Quantum Dots via an Aqueous Sol–Gel Process

**DOI:** 10.1021/acsomega.6c01552

**Published:** 2026-05-29

**Authors:** Shota Saito, Yoshiki Iso, Tetsuhiko Isobe

**Affiliations:** Department of Applied Chemistry, Faculty of Science and Technology, 12869 Keio University, 3-14-1 Hiyoshi, Kohoku-ku, Yokohama 223-8522, Japan

## Abstract

Phloroglucinol (PG)-derived graphene quantum dots (GQDs) exhibit narrow-band emission in solution; however, they suffer from severe aggregation-induced photoluminescence (PL) quenching in the solid state. Here, we report the preparation of monolithic silica nanocomposite plates incorporating GQDs via a tetramethylammonium silicate (TMAS)-based sol–gel route. Crude GQDs (C-GQDs) were synthesized through a glycothermal reaction and purified by dialysis. C-GQDs were further purified by silica-gel column chromatography to yield P-GQDs. Both C-GQDs and P-GQDs showed stable dispersion under strongly alkaline conditions owing to electrostatic repulsion between deprotonated surface hydroxy groups at the edges of GQDs. The TMAS sol–gel method enabled homogeneous GQD dispersion within silica, producing transparent plate-like monoliths that emitted blue PL at ∼470 nm. PL quantum yields of 20.4% and 23.7% were obtained at the optimal loading amounts of C-GQDs and P-GQDs, respectively. Purification enhanced spectral clarity and PL intensity, although partial PL quenching occurred during gelation due to the significant pH decrease induced by ester hydrolysis. This study demonstrates the first example of PG-derived GQDs incorporated into monolithic silica plates, providing insight into dispersion behavior, matrix-nanoparticle interactions, and remaining factors limiting PL efficiency in solid-state carbon-based nanophosphors.

## Graphical abstract

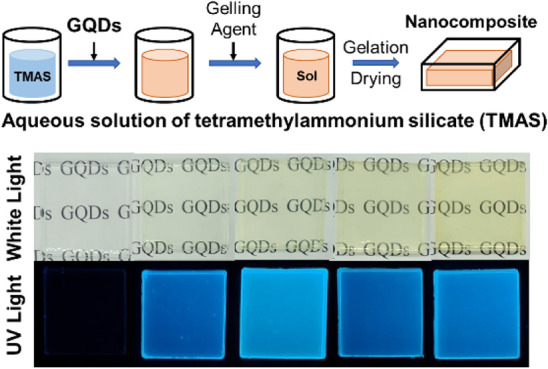

## 1 Introduction

Fluorescent graphene quantum dots (GQDs) are carbon-based fluorescent nanomaterials possessing a π-conjugated electron system.^1^ GQDs exhibit high biocompatibility, low toxicity, and low environmental impact.^2–6^ Their abundant resources and simple synthesis methods make them promising alternatives to conventional quantum dots (QDs) containing harmful metal elements like cadmium and lead, or rare metal elements like indium.^7^ Specifically, GQDs are being explored for applications in light-emitting diodes,^8–11^ bioimaging,^12–14^ metal ion sensing,^15,16^ and anticounterfeiting.^17,18^ While excellent visible-fluorescent QDs such as CsPbBr_3_ exhibit PL peaks with full width at half-maximum (fwhm) below 30 nm,^19^ many carbon-based nanomaterials show broad PL peaks.^20,21^ This leads to issues such as reduced resolution in bioimaging and diminished color purity in displays. Consequently, narrow-band fluorescent GQDs synthesized from phloroglucinol (PG), an aromatic compound containing hydroxy groups, by Yuan et al. have garnered attention.^22^ This study describes the synthesis of GQDs exhibiting diverse visible emission colors. Using ethanol (EtOH) as the solvent, GQDs with a particle size of 1.9 nm were synthesized via a solvothermal method. The resulting GQDs exhibited highly efficient narrow-band blue emission in EtOH, with a photoluminescence quantum yield (PLQY) of 66% and an fwhm of 29 nm. We have previously worked on improving the synthesis method for PG-derived GQDs.^23^ Using the glycol solvent 1,2-pentanediol and Na_3_PO_4_·12H_2_O as a catalyst in an open system promoted the dehydration condensation reaction of phloroglucinol. The yield of GQDs collected by freeze-drying after dialysis purification significantly increased from 2.8% to 99.4%. Furthermore, even after purification by silica gel column chromatography, the yield was 24.4%. The obtained GQDs had a particle size of 1.4 nm and exhibited highly efficient narrow-band emission in EtOH, with a PLQY of 75% and an fwhm of 32 nm. When the PG-induced GQDs obtained via this synthesis method were dispersed in water, they dispersed well under basic conditions and exhibited strong blue luminescence. This behavior is attributable to electrostatic repulsion caused by the negative charge of deprotonated hydroxy groups on the GQD particle surface.

The application of PG-derived GQDs in optoelectronic devices is anticipated to be a solid light-emitting layer. Aggregates and dried solid samples of GQDs do not show luminescence due to concentration quenching induced by significant fluorescence resonance energy transfer. Therefore, it is necessary to suppress GQD particle aggregation and disperse them within a matrix to create a solid light-emitting layer. The sol–gel method, which solidifies while maintaining the dispersion state of GQDs in the liquid, is considered effective for fabricating a solid light-emitting layer with dispersed GQDs. Silica is a representative material obtained via the sol–gel method; it is chemically and thermally stable and does not absorb light from the near-ultraviolet to near-infrared range. Therefore, silica excels as a dispersion matrix for nanophosphors.^24,25^

While several approaches have been reported for embedding carbon-based fluorescent nanomaterials (such as carbon dots and GQDs) into silica matrices,^24^ the resulting composites are predominantly obtained as powders, nanoparticles, or thin films coated onto substrates. Nanophosphors typically require hybridization with resins (e.g., epoxy and silicone) to be molded into macroscopic optical devices. However, such organic binders are highly susceptible to thermal degradation and photoinduced yellowing under high-power excitation sources, which severely deteriorates the long-term optical performance of the devices.^25^ We have previously developed a tetramethylammonium silicate (TMAS)-based aqueous sol–gel route to fabricate transparent monolithic silica plates embedding inorganic nanophosphors.^26–28^ The TMAS solution is a strongly basic aqueous solution containing dissolved silicate anions and tetramethylammonium cations ((CH_3_)_4_N^+^). Adding methyl lactate, an ester compound, to this solution and mixing causes a slow hydrolysis reaction. The resulting lactic acid uniformly lowers the pH of the entire solution. This protonates the silicate anions, eliminating electrostatic repulsion, and allowing network formation, leading to gelation. In the conventional sol–gel method that employs alkoxysilane, it is challenging to regulate the dispersion of nanoparticles in a mixed solvent composed of alkoxysilane, alcohol, and water. Conversely, the TMAS aqueous solution has been shown to stably disperse nanoparticles with negatively charged surfaces that exhibit a strong affinity for basic waters. For the preparation of TMAS-induced silica nanocomposites using core/shell-type InP/ZnS QDs, the QDs modified with lipophilic surface ligands were exchanged with 3-mercaptopropionic acid (MPA), a surface ligand containing carboxy groups, to hydrophilize them.^27^ The carboxy group deprotonates in basic aqueous solutions, acquiring a negative charge. Consequently, the hydrophilized QDs were dispersed well in the TMAS aqueous solution due to electrostatic repulsion. Adding methyl lactate caused gelation, yielding a plate-like QD-dispersed silica nanocomposite. The QDs maintained good dispersion, and this nanocomposite exhibited high optical transmittance. Similarly, CuInS_2_/ZnS QDs were hydrophilized to disperse in the TMAS aqueous solution, yielding a plate-like transparent fluorescent nanocomposite.^28^ When using fluorescent carbon nanomaterials with lower absorption coefficients than inorganic quantum dot phosphors, a monolithic structure can provide sufficient optical path length to compensate for the weakness in optical absorption. Building upon this established methodology, fabricating a macroscopic, highly transparent bulk composite incorporating carbon-based nanomaterials would be in demand as a next-generation optical component.

Examples of preparing solid silica-dispersing carbon-based fluorescent nanomaterials via the sol–gel method are scarce. Kwak et al. synthesized fluorescent carbon dots via the solvothermal method from 1,3-dihydroxynaphthalene in EtOH with sulfuric acid and embedded them into silica using [3-(2-aminoethylamino)­propyl]­trimethoxysilane.^21^ The resulting solid fluorescent nanocomposite lump exhibited a narrow fwhm of 31–39 nm and a high PLQY value of 78.1%. The excellent properties were achieved by encapsulating the carbon dot particles within silica while maintaining their good dispersion, thereby suppressing particle–particle proximity. This result can be attributed to the high affinity between the particles and the matrix. However, no example exists of incorporating carbon-based fluorescent nanomaterials into monolithic silica plates. TMAS-induced silica yields monolithic plate-like samples. Since PG-induced GQDs disperse well in basic aqueous solutions, they are also expected to exhibit a high affinity with TMAS aqueous solutions. No research exists on fabricating PG-induced GQD-dispersed silica nanocomposites using the sol–gel method with TMAS aqueous solutions.

This study investigates the preparation of monolithic fluorescent nanocomposites via the sol–gel method by dispersing PG-induced GQDs, which exhibit narrow-band emission, in a TMAS aqueous solution. The photoluminescence (PL) properties of nanocomposites dispersed with different amounts of GQDs are evaluated. Furthermore, GQDs are purified by using silica gel column chromatography in addition to dialysis. The PL properties of the nanocomposites are compared based on the purification state of the GQDs.

## 2 Experimental Section

### 2.1 Materials

PG (95.0%) and sodium hydroxide (NaOH; 97.0%) were purchased from FUJIFILM Wako Pure Chemical. 1,2-Pentanediol (98.0%) and methyl L-(−)-lactate were purchased from Tokyo Chemical Industry. Trisodium phosphate dodecahydrate (Na_3_PO_4_·12H_2_O; 99.0%), dichloromethane (99.5%), methanol (MeOH; 99.8%), EtOH (99.5%), and silica gel 60N (100–210 μm neutral spherical particles) were purchased from Kanto Chemical. An aqueous solution at 15–20 wt % of TMAS (99.99%) was purchased from SIGMA-Aldrich. Here, the actual solid component was 17.2 wt %, determined by drying 1.0 g of the solution at 100 °C for 1 h. All reagents were used as received without further purification.

### 2.2 Preparation of Crude GQDs

Crude GQDs were synthesized using the same method as in our previous study.^23^ PG (1.0 g, 8 mmol) and Na_3_PO_4_·2H_2_O (708 mg, 1.86 mmol) were mixed with 1,2-pentanediol (20 mL), ultrasonicated for 8 min, and heated at 180 °C for 6 h in an oil bath under airflow (300 mL min^–1^) while being stirred at 250 rpm. After air cooling to room temperature, the suspension was dialyzed in 1 L of ultrapure water using a dialysis membrane tube (3500 Da molecular-weight cutoff, 1 nm pore size) for 2 days under stirring. During the dialysis process, ultrapure water was changed three times. The electrical conductivity outside the dialysis tube decreased from 271 μS cm^–1^ to 13 μS cm^–1^ finally. The obtained residue in the tube was divided into ∼30 mL portions and transferred to each of two 100 mL egg-plant flasks. After being prefrozen at −80 °C for ∼10 min, the residue was freeze-dried at less than 20 Pa overnight to obtain crude GQD powder (C-GQDs).

### 2.3 Purification of Crude GQDs via Silica Gel Column Chromatography

Purification of the C-GQDs via silica gel column chromatography was performed using the same method as in our previous study.^23^ C-GQDs powder (30 mg) was dispersed in MeOH (2 mL) under ultrasonication and then purified with silica gel column chromatography using a dichloromethane and MeOH (6:1 v/v) eluent. The sample was divided into four fractions based on their appearance under white light and 365 nm UV light (see Figure S1). Weakly-fluorescent fraction 1 and bluish-green-emitting fraction 2 were discarded. After changing the volume ratio of the eluent to 4:1 v/v, light-green-emitting fraction 3 was discarded. The volume ratio was finally changed to 2:1 v/v to collect the green-emitting fraction 4. The solvent in fraction 4 was removed using a rotary evaporator and air-dried overnight to obtain purified GQD powder (P-GQDs).

### 2.4 Fabrication of GQDs-Silica Nanocomposite Plates

The schematic sol–gel procedure is described in Scheme 1. C-GQDs or P-GQDs powder (0–2.5 mg) was dispersed in TMAS aqueous solution (2.00 mL) under ultrasonication for 10 min. When using C-GQDs, black sediments were removed by a syringe filter with a pore size of 0.20 μm. Methyl lactate (0.14 g) was added to this dispersion and immediately stirred for >10 s. The resulting homogeneous sol was transferred to a polystyrene mold (inside dimensions: 30 × 30 × 10 mm^3^). After gelation and drying under ambient conditions for 1 day, a plate-like nanocomposite (GQDs@Silica) was obtained. Here, C-GQDs and P-GQDs-dispersed silica were named C-GQDs@Silica and P-GQDs@Silica, respectively.

**Scheme 1:**
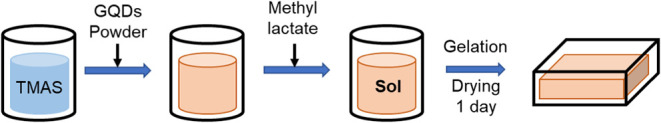
Schematic Illustration of Fabrication of GQDs@Silica

While C-GQDs contained large particles that necessitated filtration, estimation of the amount of GQDs in the resulting nanocomposite was impossible. The removal of large particles using a silica gel column enabled the estimation of the concentration of GQDs in the nanocomposite. The P-GQDs concentration in silica was estimated by the eq 1, assuming that the concentration of the TMAS solution is 15 wt %.

1 $$C=\frac{W}{0.15\times W_{S}+W}$$

where *C* is the P-GQDs concentration, *W* is the weight of the P-GQDs, and *W*_S_ is the weight of the TMAS solution.

### 2.5 Characterization

Particle sizes and morphologies were evaluated using field-emission transmission electron microscopy (FE-TEM; FEI, Tecnai 12 and Tecnai G^2^ at 120 kV and 200 kV, respectively). The samples for FE-TEM observation were prepared by drying a drop of each EtOH dispersion on a copper grid covered with an ultrathin carbon-deposited film (Oken Shoji, HRC-C10) overnight. Fourier-transform infrared (FT-IR) spectra of pressed KBr discs containing powdered samples were measured using an FT-IR spectrometer (JASCO, FT/IR-4200). Ultraviolet–visible (UV–vis) absorption spectra of dispersions and nanocomposite plates were acquired with an optical absorption spectrometer (JASCO, V-750). A film holder (JASCO, FLH-741) and an integrating sphere (JASCO, ISV-922) were used to measure in-line and total transmission spectra of nanocomposite plates, as shown in Figure S2. For dispersions, the net absorbance of GQDs was determined by subtracting the solvent absorbance. Photoluminescence (PL) and PL excitation (PLE) spectra of dispersions and nanocomposite plates were recorded by using a fluorescence spectrometer (JASCO, FP-6500). An integrating sphere (JASCO, ISF-513) was used for nanocomposite plates, as shown in Figure S3. The PLQY was determined by using a quantum efficiency measurement system (Otsuka Electronics, QE-2000–311C).

## 3 Results & Discussion

### 3.1 Evaluation of Fundamental Properties of the Prepared C-GQDs and P-GQDs

Figure 1 shows the TEM images and corresponding particle size distributions of the obtained GQDs. It should be noted that large aggregates in C-GQDs were removed using a 0.20 μm syringe filter. The average sizes of C-GQDs and P-GQDs were 9.6 ± 5.6 nm and 1.9 ± 0.6 nm, respectively. The latter value was close to the result from our previous study.^23^

**Figure 1:**
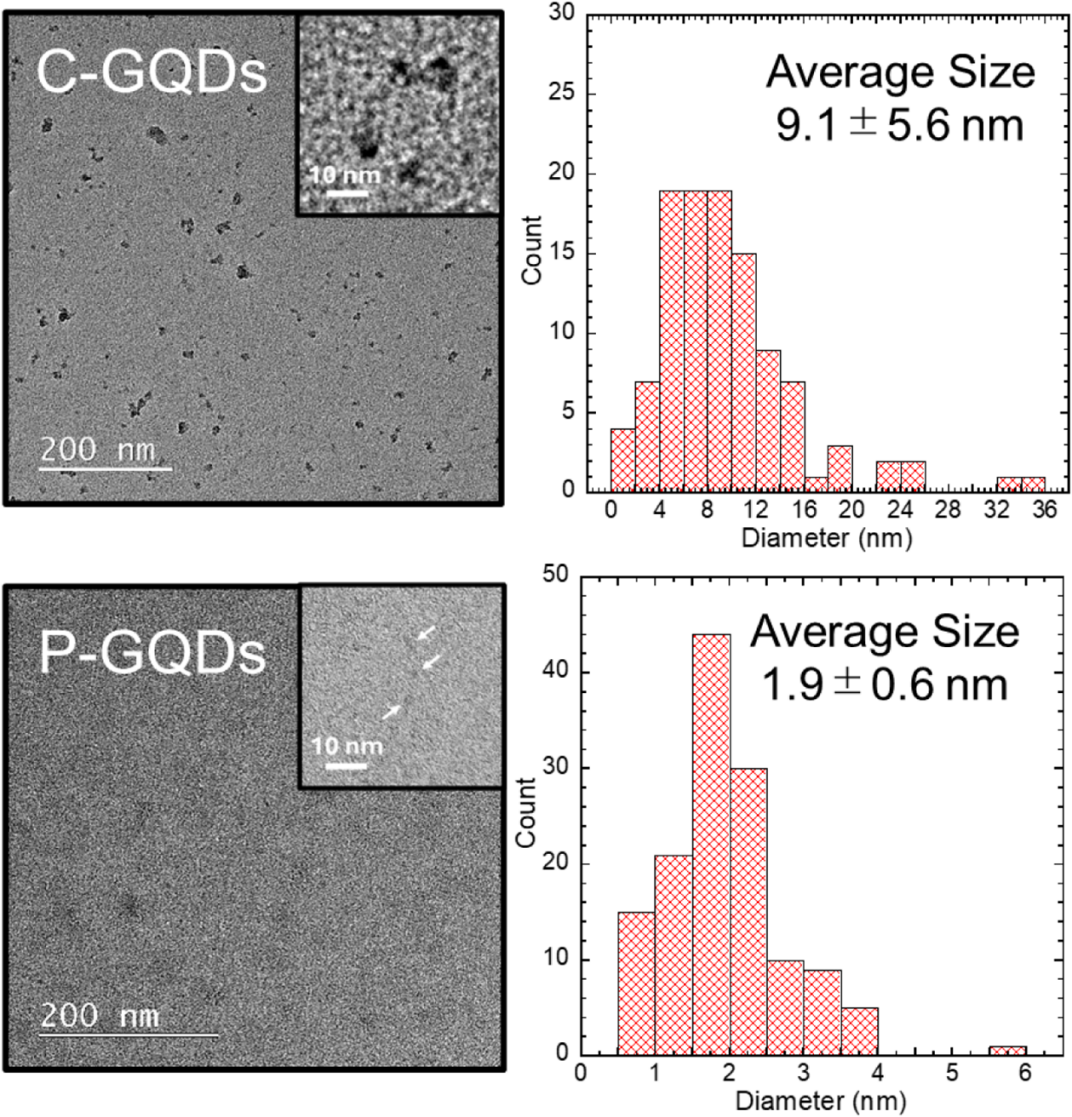
TEM images and particle size distributions of C-GQDs and P-GQDs.

Figure 2 shows the FT-IR spectra of C-GQDs and P-GQDs powders. The broad peak at 3100–3600 cm^–1^ is attributed to O–H stretching vibrations.^23^ The peaks observed around 2900 cm^–1^ correspond to C–H stretching vibrations. The sharp peak at 1750 cm^–1^ is attributed to the C=O stretching vibration, while the peak at 1600 cm^–1^ corresponds to the C=C stretching vibration. Compared to the raw PG, the C-GQDs spectrum shows significant differences, suggesting that dehydration condensation reactions proceeded in PG, altering its structure. The peak of C=O stretching vibration is possibly derived from generated C=O or COOH groups through surface oxidation during the open system synthesis. The spectrum of P-GQDs showed sharper peaks compared to C-GQDs. This indicates that impurities in C-GQDs, such as solvent molecules and byproducts, were sufficiently removed during the purification via silica gel column chromatography.

**Figure 2:**
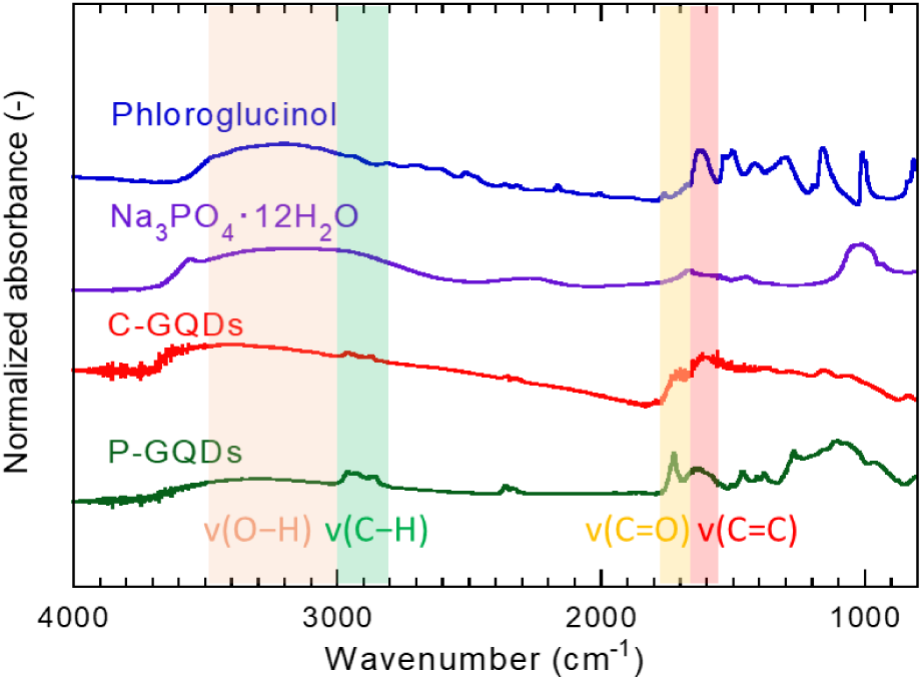
FT-IR spectra of C-GQDs and P-GQDs. The reagents PG and Na_3_PO_4_·12H_2_O are also shown.

### 3.2 Evaluation of pH Dependence on Aqueous Dispersions of C-GQDs and P-GQDs

The TMAS aqueous solution is strongly basic. The pH dependence of PL intensity for the GQD dispersions under basic conditions was evaluated in advance. C-GQDs and P-GQDs were added to water adjusted to pH 7, 9, 11, and 13 using NaOH, and were dispersed for 10 min using an ultrasonic homogenizer. Black precipitates were observed in the C-GQD dispersion; they were removed using a membrane filter (pore size 0.2 μm). The concentration was adjusted by the net absorbance to 0.05 at the optimal excitation wavelength. Figure 3 shows the changes in UV–vis absorption and PL spectra of each dispersion as a function of pH value. For C-GQDs, no significant changes in the absorption spectrum were observed with pH value. There was no distinct absorption peak, probably resulting from strong light scattering due to the aggregation of C-GQDs and overlapping absorption peaks attributed to various impurities. In contrast, an absorption peak was clearly observed at ∼440 nm for P-GQDs at pH 11 and higher. This absorption is attributed to the HOMO–LUMO electronic transition of the π-conjugated system.^23^ The elimination of impurities probably allowed the absorption peak of GQDs to appear. Conversely, no definite peak was observed at pH 9 and below, and the overall apparent absorbance was high. This would be due to strong light scattering from aggregated GQDs. These results suggest that strongly basic conditions at pH 11 and higher are necessary for stable dispersion of P-GQDs in water.

**Figure 3:**
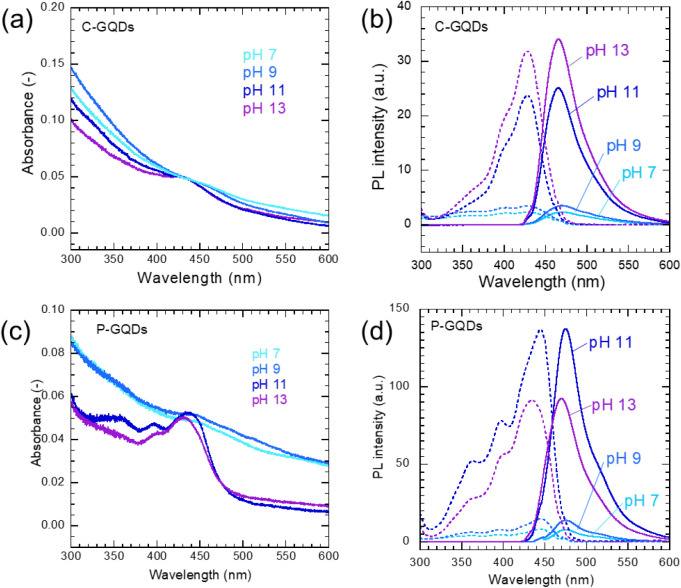
Optical spectra for (a,b) C-GQDs and (c,d) P-GQDs aqueous dispersions at various pH values. (a,c) UV–vis absorption spectra. (b,d) PLE (dashed line) and PL (solid line) spectra.

The dispersibility under basic conditions was caused by electrostatic repulsion between particles, derived from the deprotonated OH groups at the edges of GQDs.^23^ Furthermore, the observed C=O peak in the FT-IR spectrum (Figure 2) suggests that GQDs possibly possess COOH groups on their surfaces due to air oxidation. The deprotonated COOH group under strongly basic conditions would also contribute to the dispersion stability.

Aqueous dispersions of C-GQDs and P-GQDs exhibited PL peaks assigned to the π-conjugated HOMO–LUMO electronic transitions.^23^ Both PLE and PL peak wavelengths did not significantly shift, regardless of the pH value (see Table S1). The fwhm of the PL peak was above and below 50 nm for C-GQDs and P-GQDs, respectively. The decreased fwhm resulted from the purification via silica gel column chromatography. The PL intensity of both GQD dispersions increased with rising pH (see also Figure S4). The strongly basic conditions enabled the GQD particles to disperse well, suppressing concentration quenching of aggregates. The highest PL intensity for P-GQDs was approximately four times greater than that of C-GQDs. The purification process resulted in the elimination of nonfluorescent impurities, thereby enhancing the PL properties of the resulting GQDs.

### 3.3 Characterization of GQDs@Silica

TMAS-derived silica exhibited volume shrinkage and dripping during the gelation and drying process. Compared to before and after drying, the silica shrank to ∼70% of its initial volume. Nanocomposite plates of TMAS-derived silica containing C-GQDs in varying amounts were prepared. It should be noted that the actual contents of C-GQDs were lower than the amounts used as a result of large aggregates being removed via syringe filtration. Therefore, the concentration of C-GQDs@Silica was not estimated.

Figure 4 shows the appearance of the prepared C-GQDs@Silica samples under white light and 365 nm UV light irradiation. The nanocomposites were transparent and exhibited a strong brown coloration with increasing GQD amounts. Under UV light, the blank silica showed no fluorescence, while all C-GQDs@Silica samples exhibited blue luminescence. Figure 5 shows the transmission spectra. The corresponding absorption spectra are also shown in Figure S5. The in-line transmittance of the blank silica was ∼90% at 800 nm and decreased with increasing GQD amounts. This decrease is caused by the scattering of incident light from aggregated GQDs, which probably formed due to a drop in pH during gelation, resulting from weak electrostatic repulsion. To collect scattered light, total transmission spectra were measured using an integrating sphere. The blank silica exhibited ∼90% total transmittance at 800 nm. This transmittance showed little change even with increasing GQD dispersion amounts. In the total transmission spectra of all C-GQDs@Silica samples, an absorption peak attributed to GQDs was observed around 440 nm.

**Figure 4:**
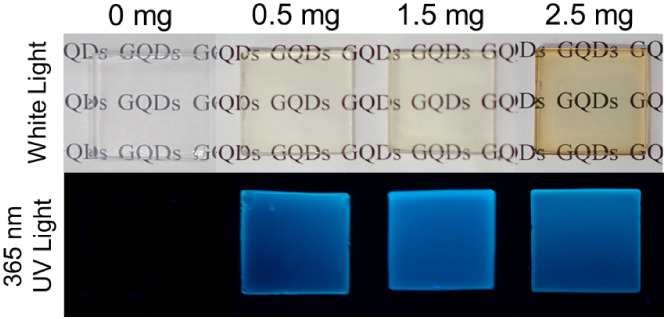
Photographs of C-GQDs@Silica under white light and 365 nm UV light.

**Figure 5:**
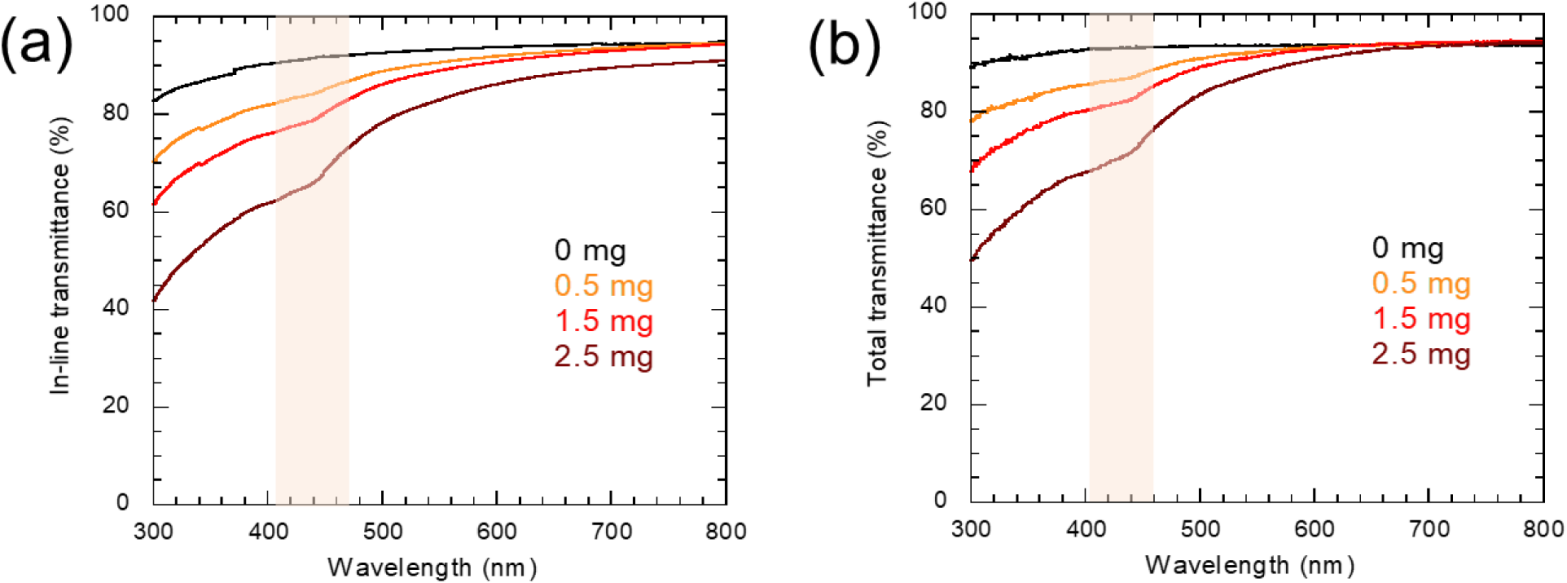
(a) In-line and (b) total transmission spectra of C-GQDs@Silica.

Figure 6 shows the PLE and PL spectra of C-GQDs@Silica. A change in the PL peak intensity was plotted in Figure S6. A PLE peak attributed to C-GQDs was observed at ∼440 nm for C-GQDs@Silica, similar to the absorption peak. The blank silica also exhibited broad and weak emission with an fwhm of 149 nm, probably due to organic residues remaining in the TMAS-derived silica. In low-concentration samples, a broad PLE peak was observed at ∼380 nm, attributed to the organic residues. The intensity of this PLE peak decreased relatively as the C-GQDs amount increased. The PL peak was observed for C-GQDs@Silica around 470 nm, which was the same as the PL peak wavelength of the C-GQD aqueous dispersions (Figure 3b and Table S1). The sharp peak at ∼460 nm should be attributed to Raman scattering of the excitation light. The fwhm of the PL peak was ∼109 nm, showing a significantly larger value compared to ∼53 nm of the dispersions. The broader PL peak would result from the overlap of the emissions of C-GQDs and residual organic matter contained within the silica mentioned above. The PL intensity increased as the GQDs amount increased up to 1.5 mg. A decrease was observed at 2.5 mg, which is attributed to concentration quenching. Therefore, the optimal amount of C-GQDs is 1.5 mg. The optimal amount of C-GQDs was therefore determined to be 1.5 mg in this work. The PLQY of C-GQDs@Silica at this amount was 20.4% (see also PL spectra for PLQY estimation in Figure S7).

**Figure 6:**
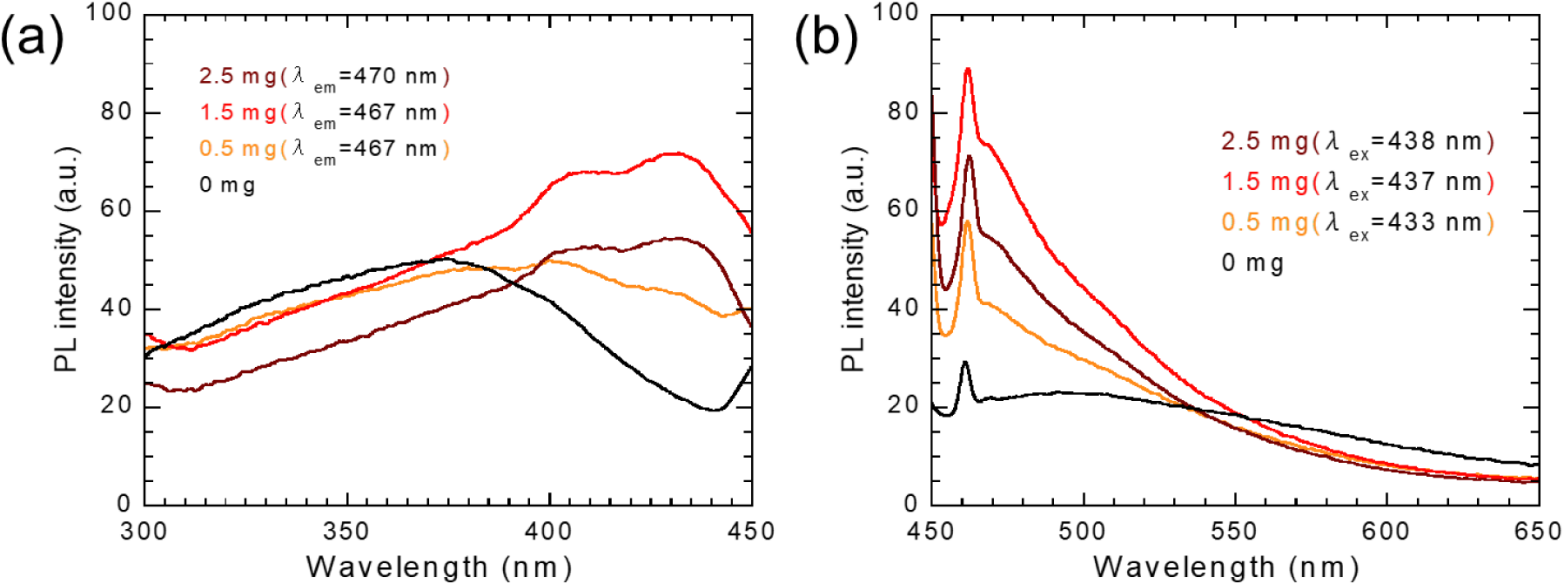
(a) PLE and (b) PL spectra of C-GQDs@Silica.

P-GQDs exhibited high dispersion in the TMAS aqueous solution, exhibiting no observable sedimentation. The filtration of aggregates, therefore, was not performed. The concentrations of P-GQDs@Silica containing 0.3, 0.5, 1.5, and 2.5 mg of GQDs corresponded to 0.05, 0.08, 0.24, and 0.40 wt %, respectively.

As shown in Figure 7, P-GQDs@Silica appeared transparent and yellow under white light and showed blue emission under 365 nm UV light. Figure 8 shows the transmission spectra. The corresponding absorption spectra are also shown in Figure S8. In the transmission spectrum, a distinct absorption peak attributed to GQDs was observed at 440 nm, resulting from the sufficient elimination of impurities via silica gel column chromatography. At 800 nm, where GQDs exhibited no absorbance, the difference between in-line transmittance and total transmittance suggests the influence of light scattering from aggregated GQDs. In-line transmittance decreased as the P-GQDs concentration increased, indicating their presence in P-GQDs@Silica.

**Figure 7:**
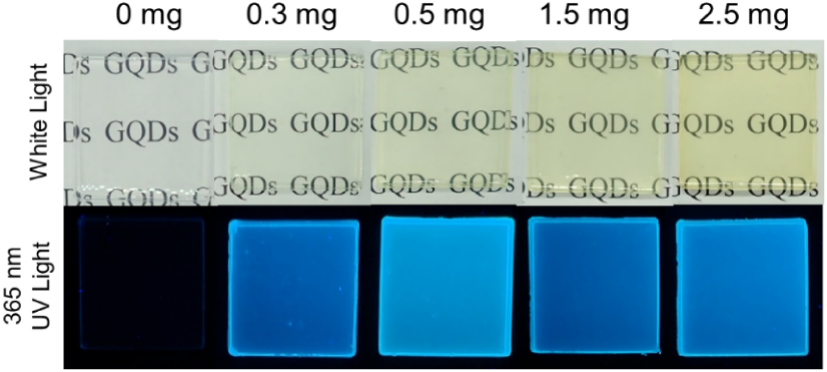
Photographs of P-GQDs@Silica under white light and 365 nm UV light.

**Figure 8:**
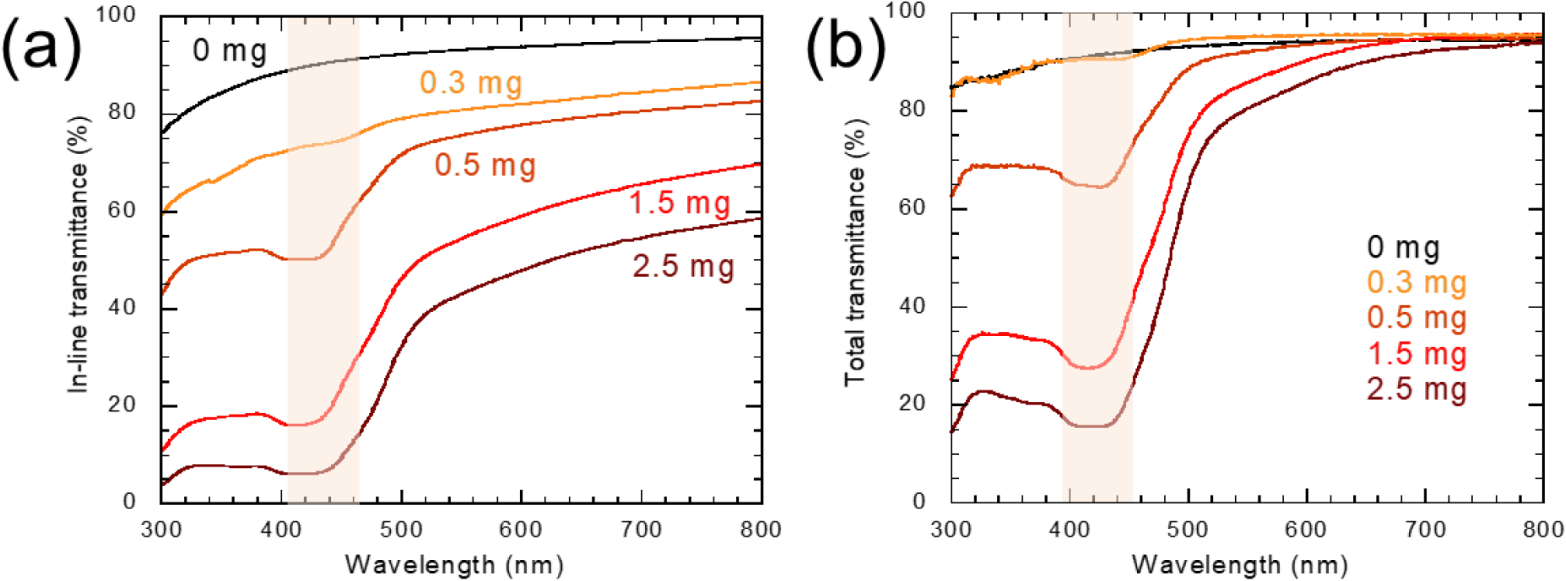
(a) In-line and (b) total transmission spectra of P-GQDs@Silica.

As shown in Figure 9, PLE and PL peaks were observed at ∼440 nm and ∼470 nm, respectively. The fwhm of the PL peak was ∼88 nm, which was larger than the 42–48 nm of the P-GQDs aqueous dispersions (Figure 3d and Table S1). This would be due to the overlap of the emission peaks attributed to P-GQDs and residual organics contained in the silica, as mentioned above. The maximum PL intensity of P-GQDs@Silica was achieved at 0.5 mg (0.08 wt %), and concentration quenching occurred at the excess concentrations. The PLQY at the optimal concentration in this work was 23.7%, which was higher than the 20.4% of the C-GQDs@Silica mentioned above.

**Figure 9:**
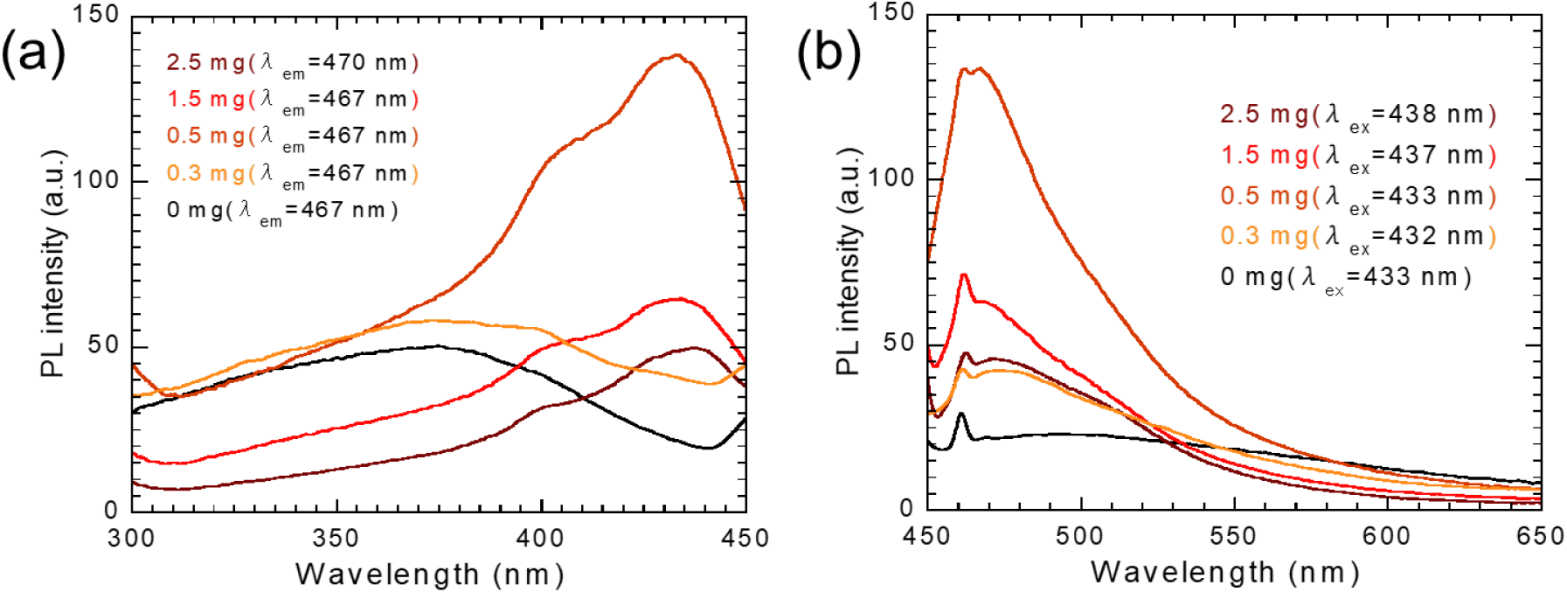
(a) PLE and (b) PL spectra of P-GQDs@Silica.

The PLQYs of C-GQDs@Silica and P-GQDs@Silica were 20.4% and 23.7%, respectively. P-GQDs dispersed in NaOH aqueous solution with pH ≥ 11, in our previous work, exhibited a PLQY > 50%.^23^ The small increase in PLQY suggests that factors other than impurities significantly reduce the PLQY. As shown in Figure 3d in the manuscript, the P-GQDs dispersion exhibited maximum PL intensity at pH 11. However, this sol–gel method, using TMAS aqueous solution, utilizes the pH decrease accompanying the hydrolysis of the ester compound. The addition of methyl lactate to the TMAS aqueous solution resulted in a rapid decrease in pH value (see Figure S9). As mentioned in Section 3.2, this may reduce the dispersion stability of GQDs. Since gelation occurred at pH ∼ 10, the pH decrease might cause PL quenching.

## 4 Conclusion

GQDs synthesized from PG were purified by dialysis and freeze-dried to obtain solid samples. The obtained C-GQDs dispersed well in aqueous solutions with high pH. This is attributed to electrostatic repulsion caused by the negatively charged OH groups at the GQD ends. Furthermore, the suppression of aggregation resulted in high fluorescence intensity. A transparent, plate-like fluorescent silica nanocomposite, C-GQDs@Silica, was prepared by dispersing GQDs in a strongly basic TMAS aqueous solution to form a sol, followed by gelation. The nanocomposite exhibited a strong brown coloration and an overall decrease in transmittance as the C-GQDs dispersion amount increased. Under excitation at approximately 430–440 nm, blue fluorescence at 470 nm was observed. This is attributed to the HOMO–LUMO electronic transition in the π-conjugated system of the GQDs. Fluorescence intensity increased with increasing C-GQD amount, but concentration quenching occurred beyond the optimal amount. P-GQDs were obtained by further purification via silica gel column chromatography. The absorption peak of P-GQDs@Silica appeared distinctly. This is thought to result from the removal of impurities exhibiting unwanted absorption during the purification process. P-GQDs@Silica also exhibited blue fluorescence from GQDs upon excitation at approximately 430–440 nm. P-GQDs@Silica showed maximum fluorescence at 0.5 mg (0.080 wt %). These results demonstrate the successful preparation of GQDs-dispersed blue-fluorescent silica nanocomposites via the sol–gel method using a TMAS aqueous solution. The PLQYs of C-GQDs@Silica and P-GQDs@Silica were 20.4% and 23.7%, respectively. The PLQY increase of only 3.3% suggests that factors other than impurities significantly reduce the PLQY. Resolving this issue in the future is expected to lead to a substantial improvement in PLQY. Moreover, by taking advantage of the unique optical properties of the carbon-based GQDs, these transparent nanocomposites hold great promise for future applications. Beyond conventional optoelectronics such as solid-state lighting, these materials could be integrated into advanced sensory architectures, including optical sensors and artificial multiperception systems for complex environmental recognition.^29–31^

## Supplementary Material



## Notes

Photographs of the silica gel column chromatography used to obtain P-GQDs (Figure S1); schematic illustration of UV–vis measurement for the nanocomposites (Figure S2); schematic illustration of PL measurement for the nanocomposites (Figure S3); PLE and PL peak wavelengths and fwhm of aqueous dispersions of C-GQDs and P-GQDs at each pH value (Table S1); changes in PL peak intensity against pH for C-GQDs and P-GQDs in aqueous dispersions (Figure S4); UV–vis absorption spectra of C-GQDs@Silica at different concentrations (Figure S5); change in PL peak intensity of C-GQDs@Silica against GQD weight used (Figure S6); PL spectra of C-GQDs@Silica and P-GQDs@Silica for PLQY estimation (Figure S7); UV–vis absorption spectra of P-GQDs@Silica at different concentrations (Figure S8); and change in pH value during gelation (Figure S9) (PDF)
