# Supplementary material for: Preparation of Monolithic Silica Nanocomposite Plates Embedding Fluorescent Graphene Quantum Dots via an Aqueous Sol–Gel Process
Source: ACS Omega. 2026 May 29;11(23):34190–8. doi: 10.1021/acsomega.6c01552 (PMC13280872; doi:10.1021/acsomega.6c01552)
Supplement: Supplementary file 1 [file ao6c01552_si_001.pdf]

Supporting Information

ACS Omega

Preparation of Monolithic Silica Nanocomposite Plates Embedding Fluorescent Graphene Quantum Dots via an Aqueous Sol-Gel Process

Shota Saito, Yoshiki Iso,* and Tetsuhiko Isobe*

Department of Applied Chemistry, Faculty of Science and Technology, Keio University, 3-14-1 Hiyoshi, Kohoku-ku, Yokohama 223-8522, Japan

*Corresponding Authors:

Yoshiki Iso – E-mail: iso@applc.keio.ac.jp; Tel.: +81 45 566 1558; Fax: +81 45 566 1551; orcid.org/0000-0001-7483-2828

Tetsuhiko Isobe – E-mail: isobe@applc.keio.ac.jp; Tel.: +81 45 566 1554; Fax: +81 45 566 1551; orcid.org/0000-0002-0868-5425

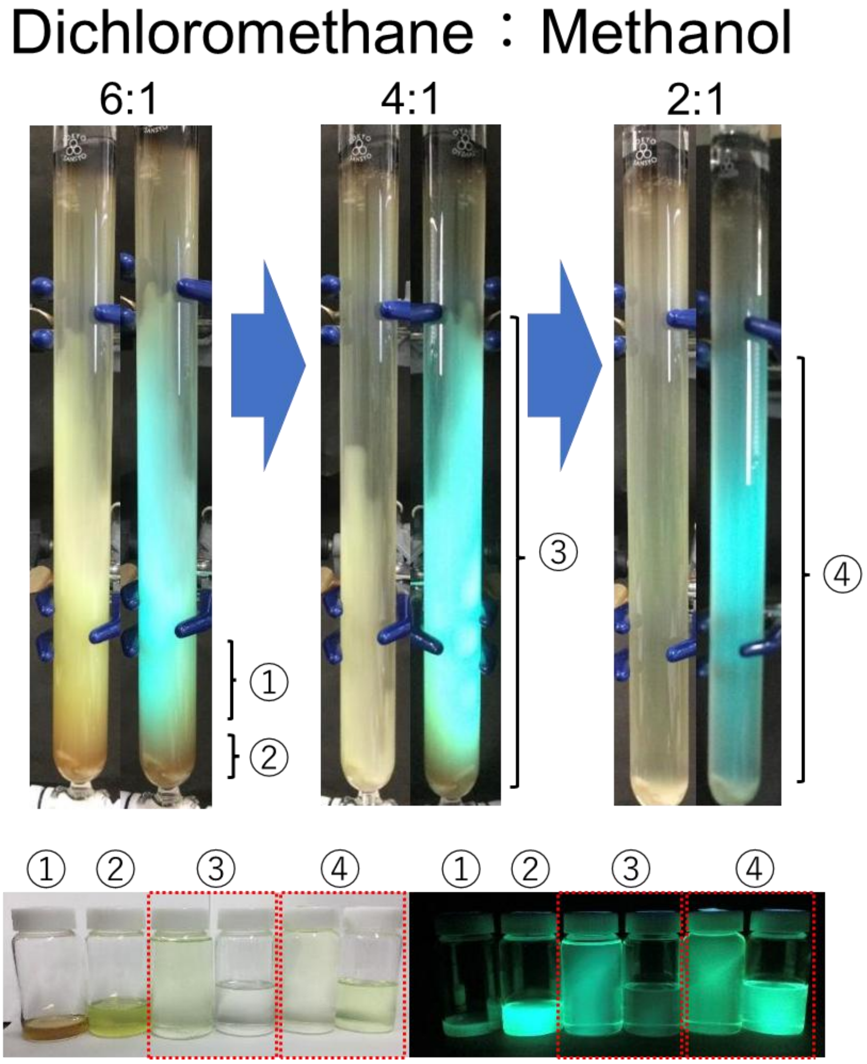

Figure S1. Photographs of the silica gel column chromatography to obtain P-GQDs under white light (left) and 365 nm UV light (right). The circled numbers indicate each fraction.

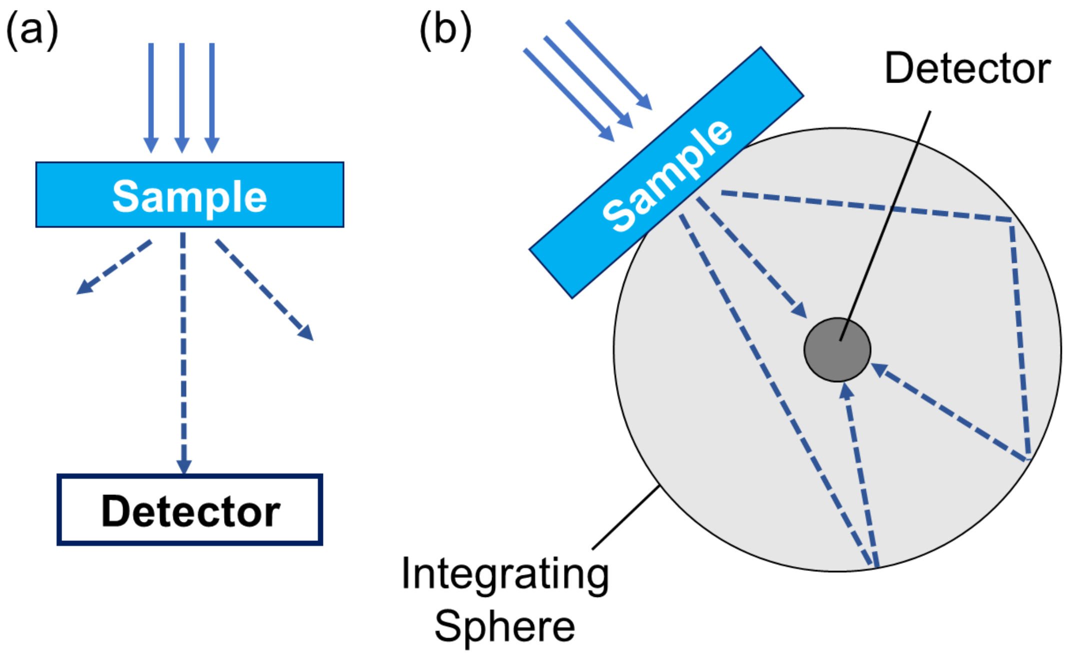

Figure S2. Schematic illustration of UV-vis measurement for the nanocomposites sample using (a) a film holder and (b) an integrating sphere.

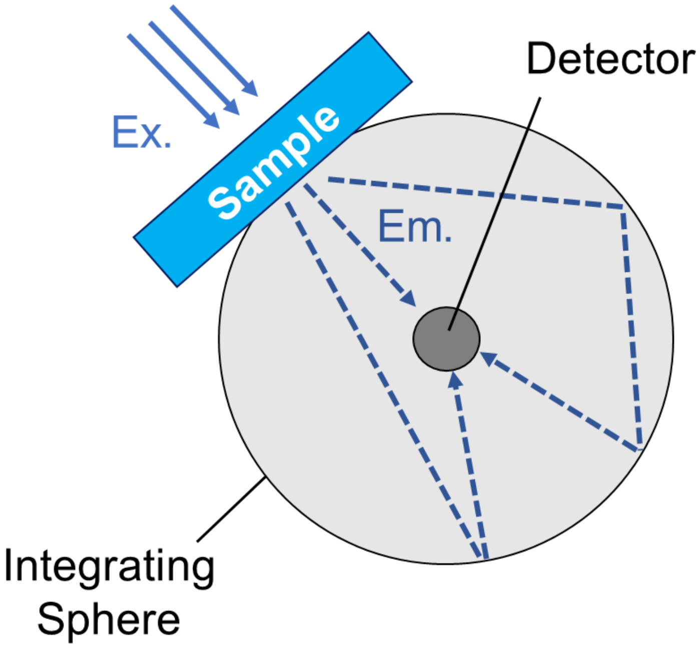

Figure S3. Schematic illustration of PL measurement for the nanocomposites sample using an integrating sphere.

Table S1. PLE and PL peak wavelengths and FWHM of aqueous dispersions of C-GQDs and P-GQDs at each pH value from Figure 3.

**Sample**		**λ_{ex} (nm)**	**λ_{em} (nm)**	**FWHM (nm)**
**C-GQDs**	pH7	427	468	77
	pH9	429	469	71
	pH11	428	464	51
	pH13	429	467	54
**P-GQDs**	pH7	444	474	42
	pH9	444	474	41
	pH11	442	473	43
	pH13	431	467	48

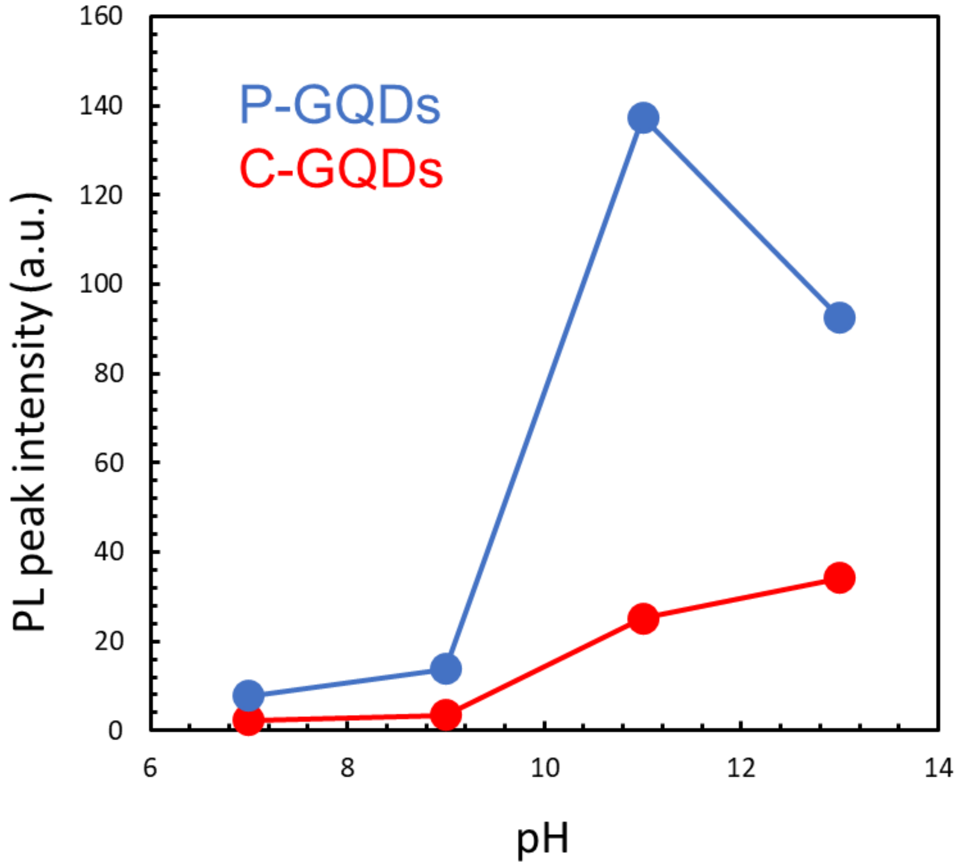

Figure S4. Changes in PL peak intensity against pH for C-GQDs and P-GQDs in aqueous dispersions.

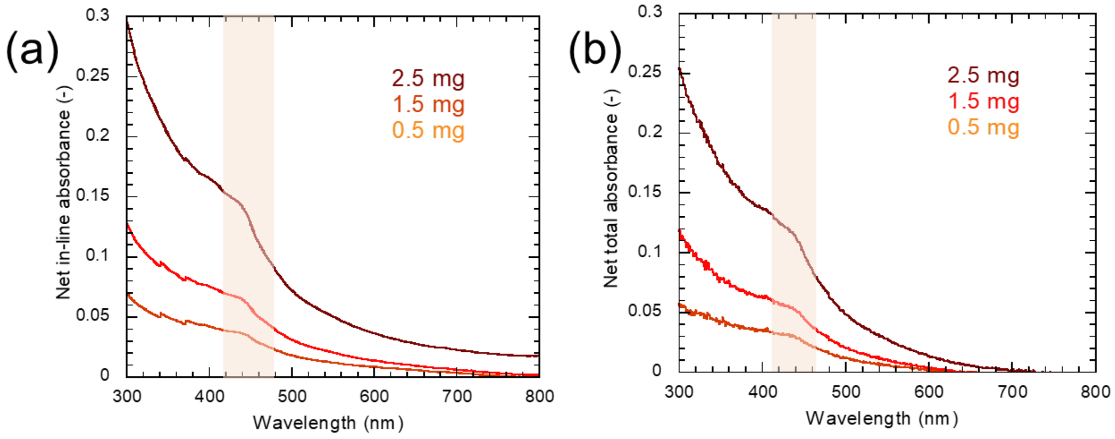

Figure S5. (a) In-line and (b) total UV-vis absorption spectra of C-GQDs@Silica at different concentrations.

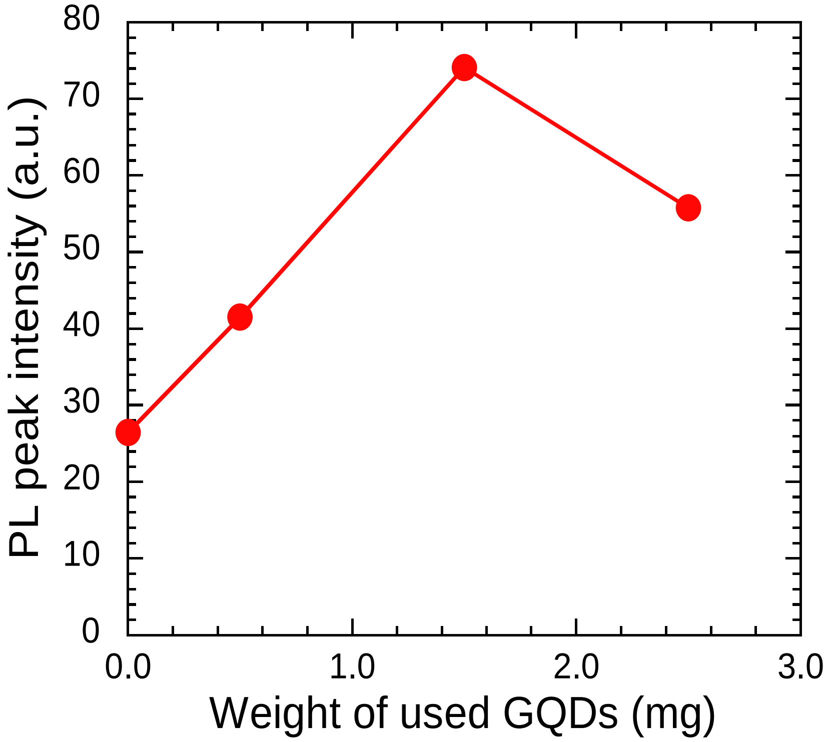

Figure S6. Change in PL peak intensity of C-GQDs@Silica against weight of used GQDs.

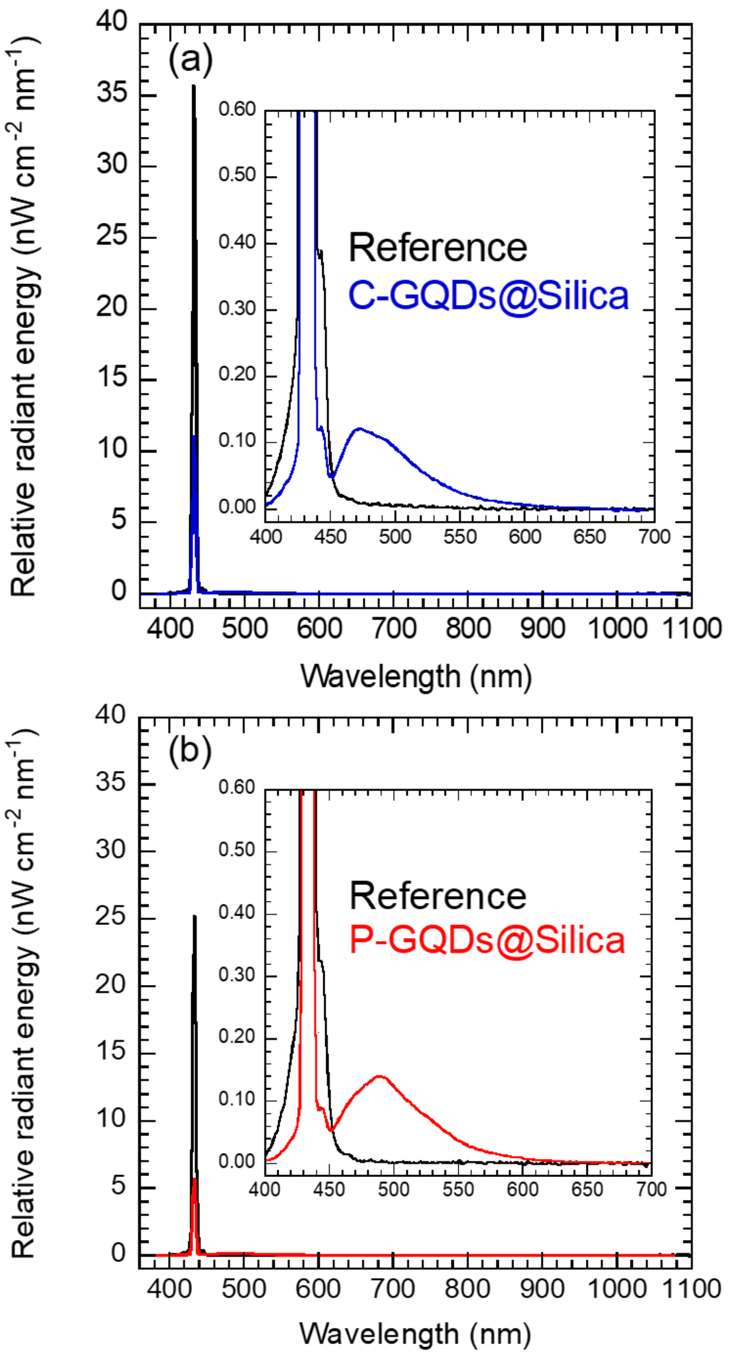

Figure S7. PL spectra of (a) C-GQDs@Silica and (b) P-GQDs@Silica for PLQY estimation. The blank silica was used as the reference.

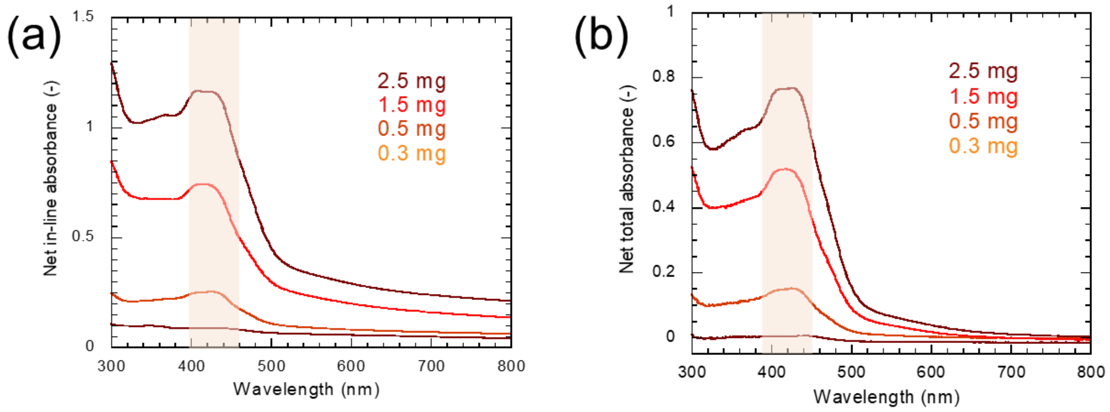

Figure S8. (a) In-line and (b) total UV-vis absorption spectra of P-GQDs@Silica at different concentrations.

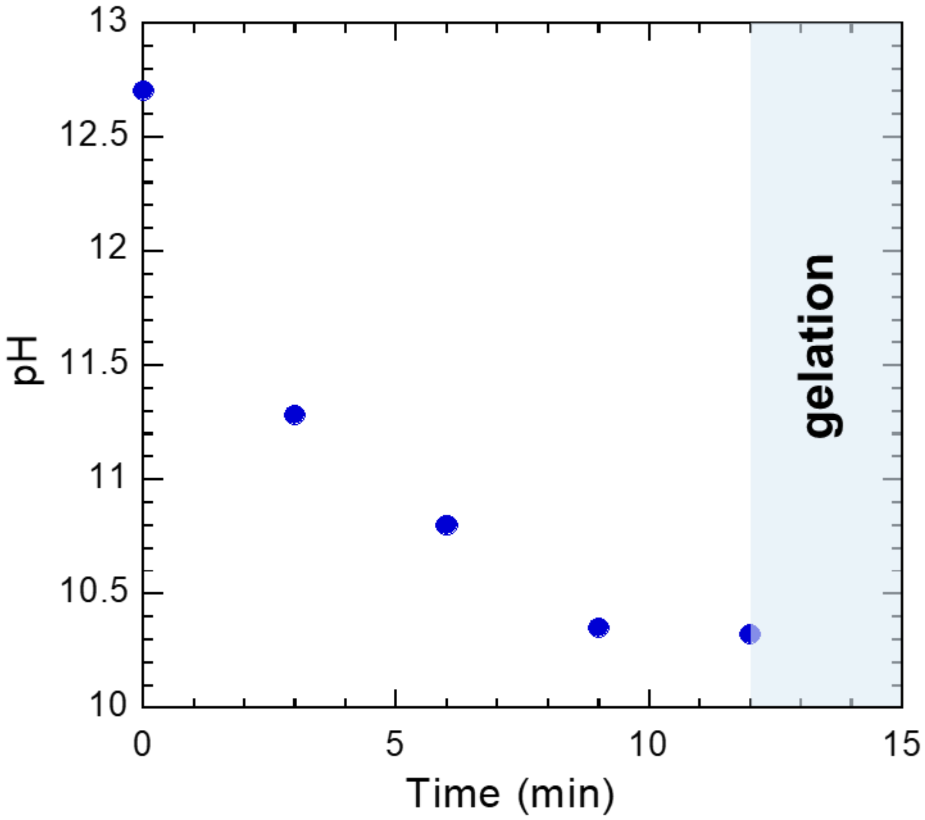

Figure S9. Change in pH value of a TMAS solution after adding the gelling agent.
